# Does the severity of neutropenia affect mortality in bacteremic cancer patients?

**DOI:** 10.1186/s12879-026-13574-2

**Published:** 2026-05-16

**Authors:** Peng-Hao Chang, Po-Yen Huang, Shian-Sen Shie, Hsuan-Ling Hsiao, Yi-Jiun Su, Chun-Fu Yeh, Ching-Tai Huang

**Affiliations:** 1 Division of Infectious Diseases, Department of Medicine, Chang Gung Memorial Hospital, 5 Fu-Shin St., Kweishan 333, Taoyuan, Taiwan; 2 Infection Control Committee, Chang Gung Memorial Hospital, Taoyuan, Taiwan; 3 Chang Gung University College of Medicine, Taoyuan, Taiwan; 4 National Tsing Hua University School of Medicine, Hsinchu, Taiwan; 5 Department of Pharmacy, Chang Gung Memorial Hospital, Taoyuan, Taiwan; 6 Department of Respiratory Therapy, Chang Gung University, Taoyuan, Taiwan; 7 Division of Hematology-Oncology, Department of Medicine, Chang Gung Memorial Hospital, Taoyuan, Taiwan

**Keywords:** Profound neutropenia, Neutropenic bacteremia, Febrile neutropenia, Prognosis, Cefepime

## Abstract

**Background:**

Concurrent bacteremia in febrile neutropenia is associated with increased mortality, but there is limited data regarding the impact of neutropenia severity on patient outcomes.

**Methods:**

A retrospective cohort study was conducted among adults (age > 18) with hematologic or solid malignancies who developed neutropenic bacteremia and received definitive cefepime treatment for > 48 h. Those who had severe neutropenia (absolute neutrophil count [ANC] 100–500 cells/µL) or profound neutropenia (ANC < 100 cells/µL) were included in the analysis. Cox proportional hazards model was used to determine the independent predictors of 30-day mortality.

**Results:**

The study included 200 bacteremias with an overall mortality rate of 13.5%. There was no difference in mortality between the profound and severe neutropenia groups (11.3% vs. 20.0%; *P* = 0.121). After adjusting for the confounding factors, profound neutropenia was not associated with 30-day mortality (adjusted hazard ratio [aHR] 0.73; 95% CI, 0.31–1.75; *P* = 0.484). Major predictors of 30-day mortality were pneumonia as the infection source (aHR 3.81; 95% CI, 1.23–11.78), higher Pitt bacteremia scores (aHR 1.26 per point), and increased Charlson comorbidity index (aHR 1.27 per point). Notably, patients with solid tumors had significantly higher 14-day mortality compared to those with hematologic malignancies (16.9% vs. 7.0%; *P* = 0.029). A relatively longer length of stay was observed in the profound neutropenia group (*P* = 0.096).

**Conclusions:**

In patients with neutropenic bacteremia treated with appropriate antibiotics, the severity of neutropenia is not associated with 30-day mortality. Our findings suggest that the potential risk associated with profound neutropenia may be mitigated by the appropriate antimicrobial therapy.

## Introduction

Febrile neutropenia (FN) constitutes a risk factor of mortality and is associated with high medical costs in patients with hematologic or solid malignancies [1, 2]. When a patient experiences FN, concurrent bacteremia further complicates the clinical course, leading to increase mortality and prolonged hospital length of stay (LOS) [3, 4].

Profound neutropenia (absolute neutrophil count [ANC] < 100 cells/µL) combined with anticipated prolonged duration (> 7 days) has been proposed as a criterion for high-risk febrile neutropenia [5]. However, whether the depth of neutropenia independently predicts mortality remains unclear. While some studies reported that ANC < 100 cells/µL is independently associated with mortality in febrile neutropenia among onco-hematological adults [6], certain investigations failed to confirm this finding [7]. The inconsistency may reflect differences in baseline patient characteristics, the critical illness, as well as the appropriateness of the selection of empiric antimicrobial agents [8, 9].

Cefepime remains a cornerstone of empirical antimicrobial therapy for FN [10]. However, given the increasing challenge of antimicrobial resistance [11], the prognostic impact of neutropenia severity may be confounded by inappropriate empirical therapy [8]. As a result, our study focuses specifically on a cohort where cefepime was initiated empirically and subsequently confirmed as definitive therapy for cefepime-susceptible bacteremia. By restricting our analysis to patients who received effective coverage, we aimed to minimize the confounding influence of antimicrobial resistance and further clarify the relationship between ANC and clinical outcomes.

## Materials and methods

### Study setting and patient selection

This retrospective cohort study was conducted between 2018 and 2022 at Linkou Chang Gung Memorial Hospital, a tertiary medical center in northern Taiwan. Subjects were identified from a database of the previous multiple-center study [12]. Hospitalized adults (age > 18 years) with bacteremia who underwent empiric cefepime for > 48 h were included. Patients were excluded if they met one of the following criteria: (1) the ANC at bacteremia onset was > 500 cells/µL, (2) no hematologic or solid malignancy, (3) bacteremia due to cefepime-nonsusceptible organisms, anaerobe, or fungi, (4) polymicrobial bacteremia, or (5) blood culture contamination, or (6) incomplete data.

### Clinical characteristics and outcomes

Medical records were reviewed for demographics, comorbid illnesses, microbiological data, antimicrobial therapy, and clinical outcomes. Details about the definitive therapy with cefepime were documented, including the time to treatment initiation, and the targeted dose was adjusted based on the estimated glomerular filtration rate. As cefepime was started empirically at the onset of bacteremia, variability in the timing of appropriate antimicrobial therapy was expected to be limited. Profound neutropenia was defined as an ANC < 100 cells/µL, and severe neutropenia was defined as an ANC 100–500 cells/µL.

Comorbid illness was assessed using the age-adjusted Charlson comorbidity index (ACCI) [13]. The source of bacteremia was determined independently by two infectious disease specialists based on chart reviews. Any disagreements were resolved by consensus. Bacteremia was deemed primary when no definite focus was found. Disease severity was evaluated at the time of bacteremia onset using the Pitt Bacteremia Score (PBS) [14]. All laboratory data were collected within 24 h of the occurrence of bacteremia.

We investigated 30-day mortality, defined as death from any cause within 30 days after the onset of the bacteremia. 14-day mortality was defined as any cause of death within 14 days after bacteremia. Clinical failure was defined as any of the following events: (1) persistent fever (body temperature > 38.0 °C) between days 3 and 7 after bacteremia onset, (2) an escalation of antimicrobial (either a switch to broader-spectrum agents or an addition of other antibiotics), or (3) Intensive care unit (ICU) admission following bacteremia. Development of recurrent bacteremia due to the same pathogen within 14 days of the initial episode was considered microbiological failure. Patients who had no clinical failure within 7 days of bacteremia onset were deemed to have a clinical response. Length of stay (LOS) was calculated from the index blood culture date to discharge from the hospital.

### Identification of bacterial species

The hospital’s laboratory used the BACTEC 9240 system (Becton, Dickinson, USA) for blood culture and matrix-assisted laser desorption ionization-time of flight (MALDI-TOF) for species identification. Antibiotic susceptibilities and minimum inhibitory concentrations (MICs) were determined using the BD Phoenix automated identification and susceptibility testing system during the study period.

### Statistical analysis

Statistical analyses were performed using the MedCalc^®^ Statistical Software version 23.3.7 (MedCalc Software Ltd, Ostend, Belgium). Univariate analyses were performed with the chi-square test or Fisher’s exact test for the categorical variables, and the Student’s t test or Mann–Whitney U test for the continuous variables, as appropriate. Survival curves were constructed and compared using the Kaplan–Meier method with the log-rank test. A multivariable Cox proportional hazards model was used to identify independent predictors of 30-day mortality. Because of limited case numbers, the model incorporated only relevant factors, including ACCI, PBS, neutropenia severity, malignancy types, and pneumonia as the source of bacteremia. The adjusted hazard ratio (aHR) and 95% confidence interval (CI) were calculated to evaluate the strength of association and the effect estimate. A P value < 0.05 was considered statistically significant.

## Results

**Fig. 1:**
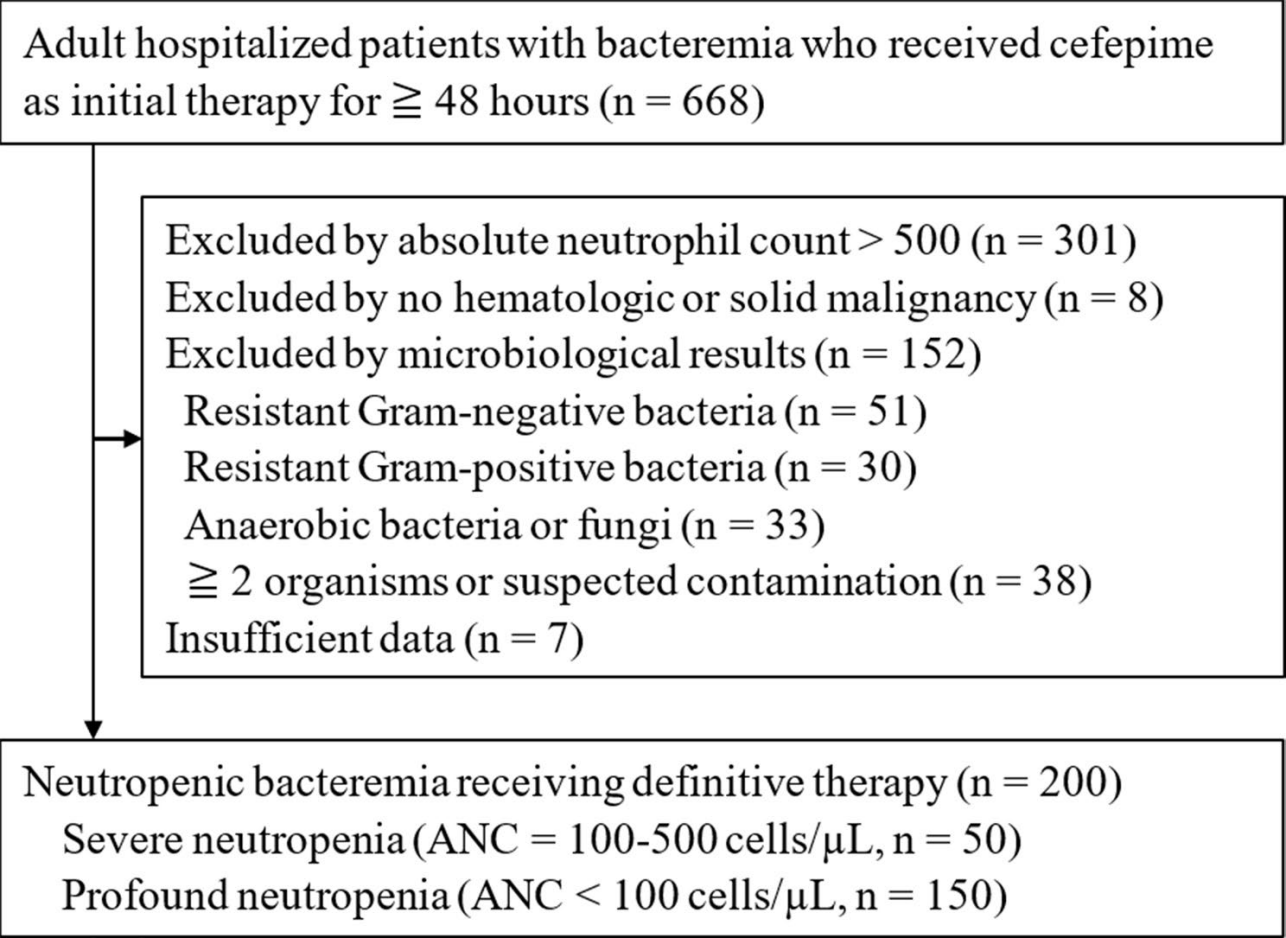
Flowchart of patient selection

A total of 200 neutropenic bacteremia episodes treated with definitive cefepime were included in the final analysis (Fig. 1).

The overall 30-day mortality rate was 13.5% (27/200). Compared to survivors, non-survivors were older and had more morbidities with higher ACCI scores. They also presented with increased PBS scores at the onset of bacteremia. Pneumonia was more frequently identified as the source of bacteremia among non-survivors (22.2% vs. 2.3%, *P* < 0.001), while primary bacteremia was more frequently observed among the survivors (Table 1).

**Table 1:** Clinical characteristics and risk factors for 30-day mortality in patients with neutropenic bacteremia receiving definitive cefepime therapy

Variables	Total^a^ (*N* = 200)	Survival (*N* = 173)	Mortality^a^ (*N* = 27)	*P* value
Sex, male	96 (48.0%)	83 (48.0%)	13 (48.1%)	0.987
Age, years	59 (44–65)	57 (47–65)	65 (58–74)	0.005
**Neutropenia**				
Profound (ANC < 100)	150 (75.0%)	133	17/150 (11.3%)	0.121
ANC, cells/µL	9 (0–30)	8 (0–30)	12 (0–35)	
Severe (ANC = 100–500)	50 (25.0%)	40	10/50 (20.0%)	0.121
ANC, cells/µL	270 (180–396)	290 (170–404)	270 (180–387)	
**Comorbidities**				
Chronic liver disease	33 (16.5%)	28	5 (18.5%)	0.762
Chronic kidney disease	13 (6.5%)	9	4 (14.8%)	0.060
Diabetes mellitus	35 (17.5%)	29	6 (22.2%)	0.489
Cardiovascular disease	15 (7.5%)	12	3 (11.1%)	0.445
Hematologic disease	129 (64.5%)	118	11/129 (8.5%)	< 0.001
Acute myeloid leukemia	83	80	3/83 (3.6%)	
Acute lymphocytic leukemia	17	16	1/17 (5.9%)	
Lymphoma	15	10	5/15 (33.3%)	
Multiple myeloma	6	6	0/6 (0.0%)	
Others	8	6	2/8 (25.0%)	
Solid Cancer	71 (35.5%)	55	16/71 (22.5%)	
Lung	11	6	5/11 (45.5%)	
Breast	11	8	3/11 (27.3%)	
Ovarian	10	9	1/10 (10.0%)	
Colon	5	3	2/5 (40.0%)	
Others	34	29	5/34 (14.7%)	
**Age-adjusted Charlson comorbidity index**	4 (3–8)	4 (3–7)	7 (5–9)	< 0.001
**Bacteremia source**				
Primary	156 (78%)	141	15/156 (9.6%)	< 0.001
Urinary tract infection	13 (6.5%)	11	2/13 (15.4%)	
Pneumonia	10 (5.0%)	4	6/10 (60.0%)	
Catheter	9 (4.5%)	8	1/9 (11.1%)	
Others	12 (6.0%)	9	3/12 (25.0%)	
**Bacterial species**				
Enterobacterales	134 (67.0%)	119	15/134 (11.2%)	0.393
Non-fermentative gram-negative bacilli	34 (17.0%)	28	6/34 (17.6%)	
Gram-positive cocci	30 (15.0%)	25	5/30 (16.7%)	
Others	2 (1.0%)	1	1/2 (50.0%)	
**Pitt bacteremia score**	1 (0–2)	1 (0–2)	1 (0–4)	0.007
**Antimicrobial therapy**				
Timely initiation^b^	169 (84.5%)	146 (84.4%)	23 (85.2%)	0.916
Targeted dose				
2 g q8h	191 (95.5%)	166	25/191 (13.1%)	0.435
< 2 g q8h	9 (4.5%)	7	2/9 (22.2%)	

Abbreviations: ANC, absolute neutrophil count

^a^ Categorical data in the Total column are presented as n (%). Categorical data in the Mortality column are presented as number of deaths/total with the 30-day mortality rate in parentheses, unless otherwise indicated. Continuous variables are expressed as median (interquartile range)

^b^ Timely initiation was defined as cefepime initiation on the same calendar day as the index blood culture. Values are expressed as n (%), with percentages indicating the proportion within each group

**Fig. 2:**
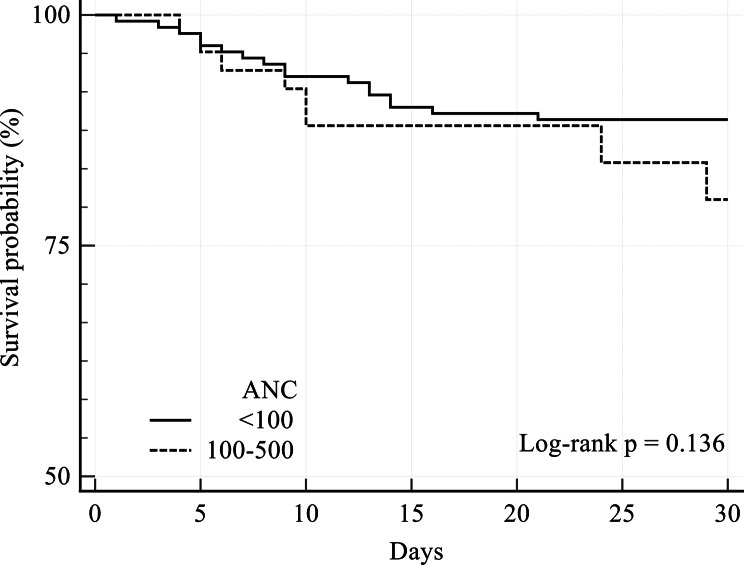
Kaplan–Meier curves of 30-day survival according to baseline absolute neutrophil count (ANC). Survival probability was compared between patients with ANC < 100 cells/µL (solid line) and ANC 100–500 cells/µL (dashed line). No statistically significant difference in 30-day survival was observed between the two groups (Log-rank *P* = 0.136)

**Fig. 3:**
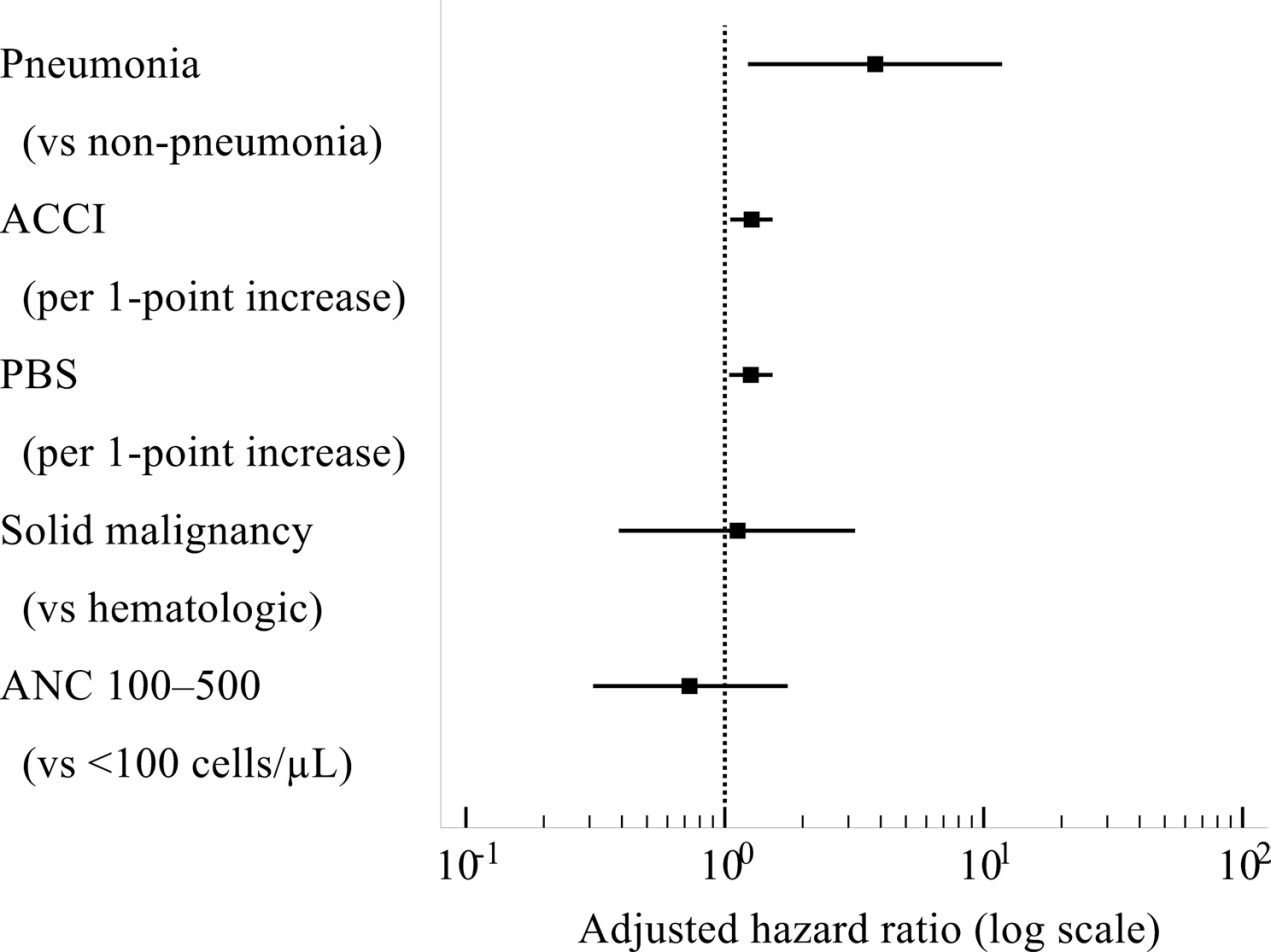
Risk factors for 30-day mortality in patients with neutropenic bacteremia: a multivariable Cox regression model. The multivariable Cox proportional hazards model was constructed using five clinical covariates: pneumonia (vs. non-pneumonia), age-adjusted Charlson comorbidity index (ACCI, per 1-point increase), Pitt bacteremia score (PBS, per 1-point increase), underlying malignancy (solid vs. hematologic), and absolute neutrophil count (ANC 100–500 vs. <100 cells/µL). Squares and horizontal lines represent adjusted hazard ratios (aHRs) and 95% confidence intervals (CIs), respectively. The vertical dashed line indicates a hazard ratio of 1.0. Independent predictors of mortality included ACCI (aHR 1.27, *p* = 0.012), PBS (aHR 1.26, *p* = 0.017), and pneumonia (aHR 3.81, *p* = 0.020). The model showed robust discrimination with a Harrell’s C-index of 0.79 (95% CI 0.72–0.87), derived from 27 deaths across 200 clinical episodes (model χ²(5) = 30.3, *p* < 0.001)

Most of the included patients had profound neutropenia (75%, 150/200). In the univariate analysis, the 30-day mortality rate was 11.3% in the profound neutropenia group and 20.0% in the severe neutropenia group (*P* = 0.121). Kaplan-Meier curves confirmed that there was no survival difference (log-rank test *P* = 0.136; Fig. 2). While profound neutropenia did not predict 30-day mortality in the Cox proportional hazards model (adjusted hazard ratio [aHR] 0.73; 95% CI, 0.31–1.75; *P* = 0.484; Fig. 3), pneumonia as the source of bacteremia, higher ACCI, and increased PBS scores were independently related to mortality. When stratified by neutropenia severity, there were no significant differences in 14-day mortality, clinical response or microbiology failure. A relatively longer length of stay was observed in the profound neutropenia group. A significantly higher 14-day mortality was associated with solid tumors, compared to hematological malignancies, despite similar rates of clinical response and microbiological failure (Table 2).

**Table 2:** Comparison of clinical outcomes stratified by the severity of neutropenia and type of malignancy

Outcome^a^	ANC < 100 (*n* = 150)	ANC 100–500 (*n* = 50)	*P* value	Hemato logic disease (*n* = 129)	Solid cancer (*n* = 71)	*P* value
14-day mortality	15/150 (10.0%)	6/50 (12.0%)	0.690	9/129 (7.0%)	12/71 (16.9%)	0.029
Microbiological failure	4/150 (2.7%)	1/50 (2.0%)	0.794	4/129 (3.1%)	1/71 (1.4%)	0.464
Clinical response^b^	100/150 (66.7%)	30/50 (60.0%)	0.393	83/129 (64.3%)	47/71 (66.2%)	0.793
LOS, days	18 (10–29)	13 (8–22)	0.096	14 (9–25)	14 (8–24)	0.616

Abbreviations: ANC, absolute neutrophil count; LOS, length of hospital stay

^a^ Categorical data are presented as n/N (%); continuous variables are expressed as median (interquartile range)

^b^ Clinical response was defined as the absence of clinical failure, where clinical failure was a composite endpoint including persistent fever during day 3–7, escalation of antimicrobial therapy, or admission to the intensive care unit

## Discussion

Studies have shown that profound neutropenia with ANC < 100 is predictive of increased mortality [6, 9]. However, this finding could be partially related to differences in the appropriateness of empiric antimicrobial therapy [8]. With the administration of appropriate antimicrobial therapy, we found that profound neutropenia did not contribute to higher 30-day mortality in cancer patients complicated with neutropenic bacteremia. In contrast, the mortality was independently associated with pneumonia, higher PBS, and higher ACCI scores. Our observation is consistent with a recent study by Calik S et al., who reported that pneumonia and shock, but not neutrophil counts, are associated with a significant risk of mortality [5].

Our findings have potential implications for antimicrobial stewardship (AMS) in supporting strategies to avoid unnecessary escalation. Clinicians often perceive profound neutropenia as a sign of frailty and tend to escalate antibiotics even when the causative bacteria are proven to be susceptible to the current regimen. In our cohort, while microbiological failure was rare, the clinical failure rate remained relatively high. This discrepancy was primarily driven by our composite definition of clinical failure, which included any antimicrobial escalation. Prior research has also shown that the implementation of AMS programs does not adversely affect clinical outcomes in high-risk hematological patients with febrile neutropenia [15–17]. Nevertheless, it is important to note that our study was not designed to compare the effectiveness of antimicrobial therapy against neutropenic bacteremia. Therefore, the decision to modify the antimicrobial regimen should be guided by disease progression, the resistance profile of the isolated bacteria, rather than solely by neutropenia severity.

We demonstrated that pneumonia is a significant risk factor for mortality in high-risk patients, a finding that aligns with prior studies [18–20]. We suspected that this high mortality seen in neutropenic pneumonia is caused by difficulties in early diagnosis and rapid progression of the disease. In FN patients, the inflammatory response could be blunted, and radiographic consolidation is often delayed [19]. The infections are also characterized by a higher bacterial burden and a rapid progression to respiratory failure or septic shock. Since pneumonia in this population may involve multidrug-resistant bacteria and polymicrobial infections, this could delay antibiotic prescription and lead to treatment failure. Our results therefore highlight that early identification of pulmonary involvement and prompt intervention are indispensable for improving survival in these high-risk groups.

Unexpectedly, a higher 14-day mortality in patients with solid tumors compared to hematological malignancies was found. In our cohort, patients with solid tumors were older (median, 64 [56–71] vs. 55 [46–65] years; *P* < 0.001) and had higher ACCI scores than those with hematological malignancies (8 [7–9] vs. 4 [2–5]; *P* < 0.001). These findings suggest that the observed difference in survival is likely driven by advanced age and underlying comorbidities rather than the type of malignancy itself. A relatively longer LOS was observed in the profound neutropenia group (*P* = 0.096). In this study, the exact duration of neutropenia for each patient could not be accurately determined, as many patients were admitted via the Emergency Department with an unknown onset of marrow suppression. However, it is reasonable to hypothesize that patients with profound neutropenia may require a longer period for hematological recovery or that clinicians decide to prolong the antimicrobial treatment out of caution. Furthermore, despite adequate control of bacteremia, these patients remain vulnerable to non-bacterial opportunistic infections, such as fungal or viral pathogens, which could further contribute to an extended LOS [21, 22].

Several limitations should be considered when interpreting our findings. First, as a single-center retrospective study involving a highly selected cohort of patients with cefepime-susceptible bacteremia, our results may not be generalizable to other clinical settings or diverse ranges of infections. Second, selection bias may exist, as clinicians may have reserved cefepime for relatively stable patients, excluding critically ill individuals who received broader-spectrum therapy. Furthermore, excluding patients with antimicrobial-resistant infections could introduce additional selection bias if the severity of neutropenia itself is a predisposing factor for such infections. Third, the relatively small sample size in the severe neutropenia group (*N* = 50) and cases with pneumonia (*N* = 10) may have limited the statistical power to detect subtle differences in mortality. Finally, our study is subject to unmeasured confounders inherent to retrospective data, such as the timing of source control or the exact duration of marrow suppression. Further multicenter, prospective studies are warranted to validate the safety of carbapenem-sparing strategies in this vulnerable population.

## Conclusion

In this retrospective cohort of patients with neutropenic bacteremia treated with appropriate antimicrobial therapy, mortality was largely determined by host factors and disease severity rather than the severity of neutropenia. Our findings suggest that the potential risk associated with profound neutropenia may be mitigated by appropriate antimicrobial therapy.

## Data Availability

The datasets analyzed during the current study are not publicly available due to institutional data governance, privacy protection, and ethical restrictions. However, de-identified data are available from the corresponding author on reasonable request.
